# Patientenerfahrung als Schlüssel zur patientenzentrierten Versorgung

**DOI:** 10.1007/s00103-025-04169-4

**Published:** 2025-12-08

**Authors:** Konstanze Blatt, Veronika Andorfer

**Affiliations:** Institut für Qualitätssicherung und Transparenz im Gesundheitswesen (IQTIG), Katharina-Heinroth-Ufer 1, 10787 Berlin, Deutschland

**Keywords:** Shared Decision Making, Qualitätssicherung, Versorgungsqualität, Patientensicherheit, Patientenbefragung, Shared decision-making, Quality assurance, Quality of care, Patient safety, Patient survey

## Abstract

Bei einer patientenzentrierten Versorgung werden die medizinischen Leistungen sowohl an klinischen Standards als auch an den Bedarfen und Bedürfnissen der Patientinnen und Patienten ausgerichtet. Die Qualitätssicherung im Gesundheitswesen zielt darauf ab, dies zu gewährleisten. Befragungen nehmen eine zentrale Rolle ein, wenn es darum geht, die Erfahrungen von Patientinnen und Patienten zu erfassen, abzubilden und zu verstehen und so eine patientenzentrierte Versorgung zu ermöglichen.

Das Institut für Qualitätssicherung und Transparenz im Gesundheitswesen (IQTIG) entwickelt im Auftrag des Gemeinsamen Bundesausschusses (G-BA) seit 2016 Patientenbefragungen als ein Element der externen medizinischen Qualitätssicherung. Bei der Entwicklung von Patientenbefragungen für 7 diagnose-/indikationsspezifische Qualitätssicherungsverfahren lassen sich folgende übergreifende Kernthemen identifizieren: Kommunikation und Interaktion (Umgang mit Patientinnen und Patienten), Entscheidungsbeteiligung und Patientenpräferenzen, Kontinuität der Versorgung und Nachsorge, Information und Aufklärung, patientenberichtete Sicherheit und Behandlungsergebnis.

Weiterhin hat das IQTIG in seinen Befragungen das Konzept des Shared Decision Making (SDM) als zentrales Element einer patientenzentrierten Versorgung in den Fragebögen verankert. Zur Operationalisierung der Qualitätsanforderungen verfolgt das IQTIG einen faktenorientierten Befragungsansatz und setzt Patient Reported Experience Measures (PREMs) und Patient Reported Outcome Measures (PROMs) ein. Die Auswertung dieser Daten liefert wichtige Hinweise auf Versorgungsdefizite und Verbesserungspotenziale, insbesondere für die Krankenhäuser und Arztpraxen, und fördert die Transparenz im Gesundheitswesen.

## Abstract (en)

Patient-centred care means aligning medical services not only with clinical standards, but also with the needs and preferences of patients. Quality assurance in the healthcare sector aims to ensure this. Surveys play a central role in capturing, reflecting and understanding patients’ experiences and needs in order to promote patient-centred care.

Since 2016, the Institute for Quality Assurance and Transparency (IQTIG) has been developing patient surveys as an element of quality assurance programmes in the German healthcare system on behalf of the Federal Joint Committee (G-BA). Throughout the development of patient surveys for seven diagnosis-/indication-specific quality assurance programmes, the following overarching core topics could be identified: communication and interaction with patients, involvement in decision-making and patient’s preferences, continuity of care and aftercare, information and education, patient-reported safety and treatment outcome.

Furthermore, the IQTIG integrated the concept of shared decision-making (SDM) as a key component of patient-centred care in its survey instruments. To operationalize the quality requirements, the IQTIG pursues a fact-based survey approach and uses patient-reported experience measures (PREMs) and patient-reported outcome measures (PROMs). The analysis of this data provides important insights into care deficits and areas for improvement especially for healthcare providers and promotes transparency in the healthcare system.

## Einleitung

Bereits 1948 hat die Weltgesundheitsorganisation (WHO) Gesundheit als dreidimensionales Konzept definiert: Neben dem körperlichen Wohlbefinden zählen demnach gleichermaßen das mentale und das soziale Wohlbefinden dazu. Mit dieser Definition erweitert sich der Fokus von der alleinigen Betrachtung geschädigter Körperfunktionen und Körperstrukturen auf die Auswirkungen dieser Schädigungen im täglichen Leben. Lebensumstände und individuelle Bedürfnisse sind neben äußerlichen Faktoren zentrale Komponenten, die das Wohlbefinden und schließlich die Gesundheit einer Person definieren.

Folglich ist eine patientenzentrierte Versorgung, die sich – basierend auf dem aktuellen wissenschaftlichen Erkenntnisstand – an den Bedarfen und Bedürfnissen der Patientinnen und Patienten orientiert, Kern einer guten Versorgungsqualität [1]. Neben dem bestverfügbaren medizinischen Wissen ist demnach versorgungsleitend, was für Betroffene wichtig ist. Was aber sind ihre Bedarfe und Bedürfnisse? Und wer stellt fest, inwiefern diese berücksichtigt wurden? Patientenzentrierung fängt bereits bei der Beantwortung dieser Fragen an. Nur die Patientinnen und Patienten selbst können benennen, was für sie wichtig ist. Gleichermaßen sind es auch sie es, die reflektieren, inwiefern ihre Bedürfnisse und Bedarfe bei ihrer Versorgung tatsächlich berücksichtigt wurden. Befragungen nehmen folglich eine zentrale Rolle ein, wenn es darum geht, die Erfahrungen von Patientinnen und Patienten zu erfassen, abzubilden und zu verstehen.

Qualitätssicherung in der medizinischen Versorgung soll sicherstellen, dass alle Patientinnen und Patienten nach dem aktuellen wissenschaftlichen Erkenntnisstand gut versorgt werden. Das fünfte Sozialgesetzbuch (SGB V) trifft hierzu Regelungen, wozu unter anderem Maßnahmen der einrichtungsübergreifenden externen Qualitätssicherung gehören (§ 135a SGB V).

Das Institut für Qualität und Transparenz im Gesundheitswesen (IQTIG) ist die verantwortliche Institution für die Entwicklung und Durchführung gesetzlich verpflichtender Maßnahmen zur Qualitätssicherung. Das IQTIG entwickelt daher seit einer Dekade Patientenbefragungen, die die patientenzentrierte Versorgungsqualität erfassen und abbilden sollen. Die patientenzentrierte Versorgung wird dabei als wesentliche Qualitätsanforderung begriffen, die sich als Leitmotiv durch den gesamten Entwicklungsprozess zieht. Aus den Ergebnissen dieser Patientenbefragungen sollen Krankenhäuser und Arztpraxen konkrete Verbesserungsmaßnahmen ableiten können.

Der Artikel gibt einen Überblick darüber, wie Erfahrungen von Patientinnen und Patienten durch die Befragungen des IQTIG erfasst werden und somit zur Förderung der patientenzentrierten Versorgung beitragen. Die Entwicklung und Umsetzung der Befragungen, beispielhafte Ergebnisse, Herausforderungen sowie Perspektiven für die externe Qualitätssicherung werden erläutert.

## Patientenbefragungen des IQTIG

Das IQTIG entwickelt im Auftrag des Gemeinsamen Bundesausschusses (G-BA) seit 2016 Patientenbefragungen als ein Element der gesetzlich verpflichtenden Qualitätssicherung. Auf Basis der Fragebögen werden Qualitätsindikatoren entwickelt, anhand derer die Qualität der Versorgung aus Patientensicht gemessen und schließlich bewertet wird.

Um dem Anspruch einer patientenzentrierten Qualitätssicherung gerecht zu werden, bindet das IQTIG bereits von Beginn an die Patientenperspektive bei der Entwicklung einer Befragung ein. So werden die Inhalte der Befragung und damit die Qualitätsanforderungen maßgeblich durch die Patientinnen und Patienten selbst bestimmt, indem deren Wünsche und Erfahrungen bezüglich ihrer medizinischen Versorgung über Fokusgruppen- und Einzelinterviews aufgenommen und systematisch ausgewertet werden.

Zudem führt das IQTIG Fokusgruppen- und Einzelinterviews mit Vertreterinnen und Vertretern der Gesundheitsprofessionen durch, die maßgeblich an der Versorgung beteiligt sind. So wird erschlossen, was ihrer Erfahrung nach für Patientinnen und Patienten wichtig ist. Die Ergebnisse dieser qualitativen Analysen werden mit den Erkenntnissen aus einer systematischen Literaturrecherche, bei der ebenfalls der Schwerpunkt auf der Patientenperspektive liegt, und einer Leitlinienrecherche zusammengeführt. Diese Wissensbestände bilden den Ausgangspunkt für die Entwicklung der Fragebögen.

Die Patientenbefragungen sind entsprechend den Qualitätssicherungsverfahren (QS-Verfahren), in die sie eingebettet sind, in der Regel indikations- oder therapiespezifisch ausgestaltet. Seit 2016 hat das IQTIG Patientenbefragungen für bereits 7 QS-Verfahren entwickelt. Eine weitere befindet sich aktuell noch im Entwicklungsprozess (Tab. 1).

**Tab. 1:** Überblick der Patientenbefragungen des Instituts für Qualität und Transparenz im Gesundheitswesen (IQTIG) und ihr Entwicklungsstatus.

Patientenbefragung/Thema	Status
Patientenbefragung *Perkutane Koronarintervention und Koronarangiografie*	Seit Juli 2022 im bundesweiten Regelbetrieb
Patientenbefragung *Schizophrenie*	Abgeschlossene Entwicklung
Patientenbefragung *Nierenersatztherapie* (Dialyse und Nierentransplantation)	Abgeschlossene Entwicklung
Patientenbefragung *Ambulante Psychotherapie*	Seit Januar 2025 im regionalen Erprobungsbetrieb
Patientenbefragung *Entlassmanagement*	Abgeschlossene Entwicklung
Patientenbefragung *Hysterektomie*	Abgeschlossene Entwicklung
Patientenbefragung *Lokal begrenztes Prostatakarzinom*	Abgeschlossene Entwicklung
Patientenbefragung *Hüft- und Knieendoprothesenversorgung*	In Entwicklung, Abschluss im Januar 2027

Im QS-Verfahren *Perkutane Koronarintervention und Koronarangiografie* (*QS PCI)* wird seit 2022 die erste Patientenbefragung der externen Qualitätssicherung bundesweit regelhaft und verpflichtend eingesetzt.^1^ Seitdem werden Patientinnen und Patienten, die mit einem Stent (perkutane koronare Intervention, PCI) versorgt werden oder eine Herzkatheteruntersuchung (Koronarangiografie) hatten, zu ihren Erfahrungen mit ihrer Versorgung befragt.

Seit 2025 erhalten Patientinnen und Patienten nach Abschluss ihrer ambulanten Richtlinien-Psychotherapie im Rahmen eines regionalen Erprobungsbetriebs in Nordrhein-Westfalen einen Fragebogen (QS ambulante Psychotherapie). Auch diese Befragung ist obligat.

## Patientenzentrierte Kernthemen in den Befragungen des IQTIG

Unabhängig von ihrer Diagnose und Behandlung werden von Patientinnen und Patienten wiederkehrend Versorgungsthemen benannt, die für sie von Bedeutung sind. So hat eine fokusgruppenübergreifende Auswertung des IQTIG gezeigt, dass sich neben Themen, die je nach Erkrankung und Behandlung variieren, 6 übergreifende Bereiche identifizieren lassen, die für Patientinnen und Patienten wichtig sind.^2^ Folgende patientenzentrierte Kernthemen finden sich in den Patientenbefragungen des IQTIG:

1. Kommunikation und Interaktion (Umgang mit Patientinnen und Patienten),
2. Entscheidungsbeteiligung und Patientenpräferenzen,
3. Kontinuität der Versorgung und Nachsorge,
4. Information und Aufklärung,
5. patientenberichtete Sicherheit,
6. Behandlungsergebnis.

Reflektiert man diese Themen vor dem Hintergrund des integrativen generischen Modells der Patientenzentrierung von Scholl et al. [2], zeigt sich trotz des unterschiedlichen Abstraktionsgrades eine starke Überschneidung. Die Dimensionen des Modells von Scholl et al. [2] adressieren gleichermaßen die Bereiche Kommunikation und Interaktion, Patientenbeteiligung, Patienteninformation, Kontinuität, individuelles Wohlbefinden und Betrachtung des Patienten/der Patientin als Individuum mit eigenen Bedürfnissen und Präferenzen.

So kann konstatiert werden, dass Patientinnen und Patienten Bereiche und Themen nennen, die Patientenzentrierung ausmachen, wenn sie nach wichtigen Merkmalen einer qualitativ hochwertigen medizinischen Versorgung aus ihrer Sicht und gemäß ihren Erfahrungen gefragt werden. Diese Kernthemen sind in allen entwickelten Patientenbefragungen des IQTIG enthalten und werden über die Fragebogenitems indikations- und behandlungsspezifisch ausdifferenziert.

Weiterhin hat das IQTIG in seinen jüngsten Entwicklungen zu Patientenbefragungen das Konzept des Shared Decision Making (SDM) als zentrales Element einer patientenzentrierten Versorgung in den Fragebögen verankert. Dabei handelt es sich um ein die Entwicklung rahmendes, übergeordnetes Konzept, das gemeinsame, gleichberechtigte Therapieentscheidungen von medizinischem Fachpersonal und Patientinnen und Patienten fördert. In Anlehnung an das 3‑Phasen-Modell von Elwyn et al. [3] sowie an das Modell von Bomhof-Roordink et al. [4] werden wesentliche Bestandteile von SDM in die Patientenbefragung aufgenommen, die sich in den Bereichen der „Information und Aufklärung“, „Entscheidungsbeteiligung und Patientenpräferenzen“ sowie „Kommunikation und Interaktion“ widerspiegeln.

Die Patientenbefragungen des IQTIG erfassen u. a., inwiefern Patientinnen und Patienten Informationen zu möglichen Behandlungsoptionen mit den damit verbundenen positiven und negativen Konsequenzen erhalten haben, sodass sie zu einer Entscheidung bei der Therapiewahl befähigt werden. Auch Bedenkzeit sowie individuelle Bedürfnisse und Präferenzen bei der Therapiefindung werden berücksichtigt. Eine patientenzentrierte Kommunikation ist ein weiterer Bestandteil, der in diesem Zusammenhang erfasst wird. Hier geht es um die sprachliche und inhaltliche Nachvollziehbarkeit der vermittelten Informationen, aber auch darum, inwiefern ein wertschätzendes und respektvolles Verhalten des medizinischen Personals sowie eine zugewandte Gesprächsatmosphäre gegeben waren.

## Faktenorientierter Befragungsansatz

Bei der Operationalisierung der zu Beginn des Entwicklungsprozesses identifizierten Qualitätsanforderungen verfolgt das IQTIG einen faktenorientierten Befragungsansatz und setzt Patient Reported Experience Measures (PREMs) und Patient Reported Outcome Measures (PROMs) ein. Kern dieses Befragungsansatzes ist, dass Patientinnen und Patienten als „Zeugen“ ihre Behandlung nach dem Eintreten bzw. Nichteintreten konkreter qualitätsrelevanter Situationen, Ereignisse und Zustände gefragt werden [5–8].

Dieser Befragungsansatz eignet sich insbesondere für die Qualitätssicherung: Die Qualitätsanforderungen an eine hochwertige patientenzentrierte Versorgung werden ähnlich einer Checkliste über die Fragebogenitems geprüft. So ermöglicht die patientenzentrierte Erfassung von Qualitätsanforderungen eine direkte Verbindung mit den im Praxisalltag etablierten Prozessen und Strukturen. Krankenhäuser und Arztpraxen erhalten anhand der Ergebnisse der Patientenbefragung über die Qualitätsindikatoren mit ihren zugehörigen Items konkrete Ansätze für entsprechende Verbesserungsmaßnahmen.

Die Befragungen des IQTIG verfolgen damit eine patientenzentrierte Ausrichtung in zweifacher Hinsicht: Zum einen werden durch den Einbezug der Betroffenen selbst Qualitätsanforderungen definiert, die ihren Bedarfen und Bedürfnissen entsprechen. Diese Anforderungen werden zum Inhalt einer Patientenbefragung, die wiederum Grundlage für die Bewertung der Versorgungsqualität anhand von Qualitätsindikatoren ist und damit Startpunkt für die Umsetzung entsprechender Verbesserungsmaßnahmen. Dies trägt zum anderen zur Etablierung und Sicherung einer patientenzentrierten Versorgung in der Praxis bei.

## Beispielhafte Ergebnisse der Patientenbefragungen des IQTIG

Wie relevant die patientenzentrierte Ausrichtung der Befragungen ist, zeigen u. a. die aktuellen Auswertungen zu der im Regelbetrieb etablierten Patientenbefragung des Verfahrens *QS PCI.* Im Jahr 2024 wurden bundesweit insgesamt 135.884 Fragebögen verschickt [9].^3^ Mit einer Rücklaufquote von 52,33 % gingen 71.106 Fragebögen in die Auswertungen ein. Die Ergebnisse geben Einblick in den von Patientinnen und Patienten erlebten Versorgungsalltag von 1047 Arztpraxen und Krankenhäusern, die PCI oder Koronarangiografien durchführen.

So wird beispielsweise deutlich, dass viele Patientinnen und Patienten, die einen Stent erhalten, zuvor zu wenig über mögliche Behandlungsalternativen informiert werden. Auch zeigt sich, dass Patientinnen und Patienten zwar meist über die Art, Dosierung und Dauer der Medikamenteneinnahme informiert werden. Allerdings werden weiterreichende, relevante Informationen zu möglichen Neben- und Wechselwirkungen und zur Medikamentenunterbrechung deutlich seltener gegeben.

Anhand von 2 Qualitätsindikatoren der Patientenbefragung *QS PCI* lässt sich exemplarisch aufzeigen, wie die detaillierte Darstellung der Befragungsergebnisse konkrete Hinweise gibt, welche der patientenrelevanten Erklärungen laut Rückmeldung der Betroffenen in den Krankenhäusern und Arztpraxen unzureichend kommuniziert wurden.

### Beispielhafte Auswertungen zur Patientenbefragung QS *PCI* für die Qualitätsindikatoren 56106 und 56107 (Quelle: [9, S. 73])

Mit dem Indikator 56106 *Information vor der elektiven Prozedur* werden über die Patientenbefragung folgende Themen erfasst:

- Information zu Behandlungsalternativen,
- Information zur therapeutischen Konsequenz (regelmäßige Medikamenteneinnahme nach einem Stent),
- Information zum Ablauf der Prozedur, einschließlich zu erwartender Schmerzen,
- Information zur Möglichkeit, dass bei einer Koronarangiografie gleich ein Stent gesetzt werden kann (einzeitiger Eingriff),
- Information zu der Möglichkeit, auf Wunsch eine Sedierung zu erhalten,
- Information zum Ablauf der Nachbeobachtung,
- Möglichkeit, über Sorgen und Ängste zu sprechen,

Die Indikatorwerte sind als Indexwerte aus den zugeordneten Fragebogenitems angelegt (maximaler Wert: 100). Auf der positiv gepolten Skala von 0 bis 100 konnte ein Durchschnittswert von 75,09 Punkten erreicht werden (Min.: 34,11, Max.: 95,79; *N* = 48.610 Patientinnen und Patienten von 858 Leistungserbringern [9, S. 73]). Bei der genaueren Analyse der Antwortverteilungen der zugrunde liegenden Items zeigt sich, dass vor allem zu wenig über Behandlungsalternativen gesprochen wird. So gaben 43,64 % (*n* = 21.213) der befragten Patientinnen und Patienten an, vor der Stenteinlage nicht darüber informiert worden zu sein, dass es noch andere Behandlungsmöglichkeiten gibt [9, S. 73].

Über den Indikator 56107 *Informationen zum Absetzen oder Umstellen der Medikamente nach einer PCI* wird konkretes Verbesserungspotenzial deutlich, das ganz praktische Implikationen für den Patientenalltag nach der Versorgung haben kann. Mit dem Indikator werden Themen angesprochen wie:

- Informationen zur Dauer der Medikamenteneinnahme,
- Informationen zu Nebenwirkungen und Wechselwirkungen von Medikamenten,
- empfohlenes Verhalten bei geplanter und ungeplanter Therapieunterbrechung der Medikation,
- Informationen zu Art und Dosierung von Medikamenten.

Auf Bundesebene wurde ein Durchschnittswert von 61,62 Punkten erreicht (Min.: 29,32, Max.: 88,37; *N* = 21.614 Patientinnen und Patienten von 749 Leistungserbringern [9, S. 73]).

Die genauere Betrachtung verdeutlicht, dass Patientinnen und Patienten meist über die Art, Dosierung und Dauer der Medikamenteneinnahme informiert werden. Weiterreichende Informationen werden deutlich weniger gegeben: Über die Hälfte der Befragten (58,20 %, *n* = 12.580) verneinten die Frage, ob sie darüber informiert wurden, welche möglichen Neben- und Wechselwirkungen die Medikamente haben können [9, S. 73]. Ähnlich viele Patientinnen und Patienten gaben an, keine Information zum Vorgehen erhalten zu haben, wenn sie vergessen haben, das Medikament einzunehmen (58,08 %, *n* = 12.554; [9, S. 73]).

## Impact für den Versorgungsalltag

Die Patientenbefragung als ein Instrument der Qualitätssicherung ist auf die aggregierte Auswertung und Ergebnisinterpretation ausgerichtet. Die Ergebnisse geben das Erleben der befragten Patientinnen und Patienten eines Krankenhauses oder einer Arztpraxis wieder. Sie beziehen sich auf qualitätsrelevante Prozesse, prozessnahe Strukturen und den Gesundheitszustand der Patientinnen und Patienten. Vor allem PROMs werden in anderen Kontexten häufig für eine patientenindividuelle Therapieplanung und Ergebnismessung eingesetzt [10]. Diese individuelle Auswertung ist nicht Fokus der externen Qualitätssicherung. Auf Basis der aggregierten Rückmeldungen als ein „durchschnittliches Patientenfeedback“ sollen hier vielmehr Verbesserungsmaßnahmen abgeleitet werden, die die grundsätzliche Ausgestaltung des Versorgungsalltags betreffen.

Entsprechend erhalten die Krankenhäuser und Arztpraxen in aufbereiteter Berichtsform ihre Ergebnisse der Befragung. Vor Ort müssen die Strukturen und Prozesse identifiziert werden, die mit den erfragten Inhalten des Fragebogens in Verbindung gebracht werden. So kann im nächsten Schritt erkannt werden, inwiefern diese Strukturen und Prozesse entsprechend optimiert werden können. Der Umgang mit den Ergebnissen folgt also 3 Schritten: die Analyse der Ergebnisse der Patientenbefragung (Ergebnisanalyse), die Analyse des Versorgungsalltags (Praxisanalyse) und die Ableitung von Verbesserungsmaßnahmen. Dieses Vorgehen soll an einem Beispiel veranschaulicht werden:

Ein wichtiges Element insbesondere von SDM ist die verständliche Kommunikation von Behandlungsalternativen. So könnten Patientinnen und Patienten über die Befragung beispielsweise angeben, dass sie unzureichend oder gar nicht über Behandlungsalternativen informiert wurden. Unabhängig davon, ob diese Informationen tatsächlich nicht gegeben oder aber so vermittelt wurden, dass sie von den Patientinnen und Patienten nicht verstanden oder erinnert werden konnten: Dem patientenzentrierten Ansatz folgend soll das Ergebnis der Patientenbefragung Anlass sein, genauer zu betrachten, ob und wie diese Informationsgabe in der Regel erfolgt. Gibt es ein Standardprozedere, das sicherstellt, dass Patientinnen und Patienten diese Informationen zuverlässig erhalten? Wann, durch wen und in welcher Form werden die Informationen gegeben? Anhand dieser Analyse können Möglichkeiten für Verbesserungsmaßnahmen abgeleitet werden, die darauf abzielen, dass die relevanten Informationen zuverlässig so vermittelt werden, dass die Patientinnen und Patienten sie in der Regel verstehen und erinnern.

So könnte es beispielsweise sinnvoll sein, eine Checkliste für Informations- und Aufklärungsinhalte einzuführen, die das Besprechen bestimmter Themen jenseits der standardisierten Aufklärungsbögen sicherstellt. Auch kann die systematische Implementierung von Kommunikationsstrategien bei der Informationsgabe, beispielsweise der „Teach-Back-Methode“, zu einer Verbesserung führen. Kern dieses Ansatzes ist, dass Patientinnen und Patienten die gegebene Information paraphrasieren und gezielte Rückfragen stellen, bis der Informationsinhalt wie intendiert verstanden wurde, was nachweisliche Effekte auf das Verstehen und Erinnern der Information hat [11].^4^

## Herausforderungen

Die erfolgreiche Implementierung einer Patientenbefragung als regelhaftes Instrument der externen Qualitätssicherung, die eine patientenzentrierte Versorgung fördern soll, ist mit Herausforderungen verbunden.

Fragebogenlogistik und Datenflüsse erfordern eine entsprechende Infrastruktur, die etabliert werden muss. Um den datenschutzkonformen Ablauf der anonymen Befragung nach Abschluss der jeweiligen medizinischen Versorgung zu gewährleisten, wurde beispielsweise eine zentrale Versendestelle für die Patientenbefragung eingerichtet. Diese ist vom G‑BA beauftragt und für die Stichprobenziehung sowie für den Druck und Versand der Unterlagen von Patientenbefragungen zuständig. Die ausgefüllten Fragebögen werden von den Patientinnen und Patienten an eine zentrale, ebenfalls eigens dafür geschaffene Fragebogenannahmestelle geschickt, die im Auftrag des IQTIG für die Annahme und Verarbeitung der Daten zuständig ist.

Auch erhält eine Patientenbefragung durch eine deutschlandweite, regelhafte Umsetzung einen Umfang und eine Komplexität, die es kontinuierlich für alle Beteiligten zu bewältigen gilt. Hier sind nicht zuletzt gute Softwareprodukte gefragt, die aufwandsarm und zuverlässig die erforderlichen Prozesse wie Dateneingabe und -weiterleitung unterstützen.

Grundsätzlich müssen die Ergebnisse der Patientenbefragung belastbar sein, um tatsächliche Konsequenzen daraus abzuleiten. Aussagekräftige Rücklaufquoten und eine gute Entwicklungs- und Auswertungsmethodik sind dafür unabdingbar. Deshalb hat sich das IQTIG zu hohen methodischen Standards verpflichtet, was sich in einem intensiven, mehrstufigen Entwicklungsprozess unter Einbindung externer fachlicher Expertise ausdrückt [12].

Die oben berichtete aktuelle Rücklaufquote der Patientenbefragung *QS PCI* von über 50 % liegt weit über den üblicherweise berichteten Rücklaufquoten in Bevölkerungsumfragen [13, 14]. Dies zeigt nicht nur das hohe Interesse und die Bedeutung, die die Befragung für die Patientinnen und Patienten hat. Die hohe Rücklaufquote verweist auch auf eine solide Datengrundlage.

Damit die Ergebnisse der Patientenbefragung auch zu einer besseren, patientenzentrierten Versorgung führen, ist insbesondere in den versorgenden Krankenhäusern und Arztpraxen eine Auseinandersetzung hiermit notwendig. Dies erfordert Zeit, die Fähigkeit, Ansatzpunkte im Versorgungsalltag zu erkennen, und die Bereitschaft für Veränderung.

## Ausblick und Perspektiven

Mithilfe des faktenorientierten Befragungsansatzes kann mit den Patientenbefragungen des IQTIG und damit der externen Qualitätssicherung erkannt werden, an welchen entscheidenden Stellen die Versorgung im Sinne der Patientenzentrierung verbessert werden kann. Deshalb ist es wichtig, dass sich dieses Instrument weiter etabliert.

Um die systematische Erfassung der Patientenperspektive als „Motor“ einer patientenzentrierten Versorgung zu verbreiten, wäre eine allgemeine Patientenbefragung sinnvoll, die über die externe Qualitätssicherung etabliert wird. Mit einer solchen indikations- und therapieübergreifenden Befragung können allgemeine Qualitätsanforderungen thematisiert werden, die sich an den Kerndimensionen einer patientenzentrierten Versorgung orientieren. Diese Kerndimensionen sollten sich primär auf Prozesse und Strukturen der Versorgung beziehen und über PREMs abgebildet werden, z. B. generelle Informationen zu Behandlungsalternativen, Entscheidungsbeteiligung, Schmerzmanagement, Entlassung und Weiterversorgung.

Eine solche allgemeine Basisbefragung sollte um verfahrensspezifische Patientenbefragungen ergänzt werden, um ganz gezielt Qualitätsanforderungen bestimmter Versorgungsbereiche und deren Besonderheiten betrachten zu können. Damit lassen sich insbesondere indikations- und versorgungsspezifische Outcomes (PROMs) aufgreifen, die in einer allgemeinen Patientenbefragung nicht erfasst werden können.

Die Patientenbefragungen des IQTIG werden als selbstadministrierte Paper-Pencil-Befragungen durchgeführt. Hierzu wird den Patientinnen und Patienten nach Abschluss ihrer Versorgung ein Fragenbogen zu geschickt. Perspektivisch sollen die Befragungen um einen onlinebasierten Modus erweitert werden, der auch barrierefrei ausgestaltet werden soll. Die ersten Entwicklungen dazu laufen für die Patientenbefragung *QS PCI*.^5^

Damit die Versorgenden aus den Befragungsergebnissen zielgerichtet und praxisnah Erkenntnisse für mögliche Verbesserungsmaßnahmen ableiten können, sollten diese entsprechend aufbereitet sein. Bislang werden die Ergebnisse in Berichtsform als PDF-Dokument kommuniziert. Eine anschaulichere Aufbereitung könnte beispielsweise durch ein digitales Dashboard ermöglicht werden, das als Heatmap zur Darstellung der Befragungsergebnisse genutzt werden kann. In Verknüpfung mit einem etablierten Qualitätsmanagement können so mögliche Schwachstellen schnell erkannt werden und Verbesserungsmaßnahmen eingeleitet werden. Dies gilt es für die Ergebniskommunikation an die leistungserbringenden Einrichtungen im Rahmen der externen Qualitätssicherung zu etablieren.

## Fazit

Patientenzentrierung, einschließlich SDM, sind als Leitprinzipien der medizinischen Versorgung essenziell. Therapieentscheidungen begleiten Patientinnen und Patienten mit ihren Konsequenzen im positiven wie im negativen Sinn oftmals ein Leben lang. Fehlende, nicht verstandene oder nicht erinnerbare Informationen führen im besten Fall nur zur Verunsicherung der Patientinnen und Patienten, im schlechtesten Fall gefährden sie das Patientenwohl.

Die Entwicklungsmethodik des IQTIG ermöglicht es, Befragungen einzusetzen, die Qualitätsanforderungen adressieren, die für Patientinnen und Patienten von Bedeutung sind und die zentrale Merkmale einer patientenzentrierten Versorgung darstellen, die gleichermaßen von ihnen selbst definiert sind.

Zugleich erfolgt über die Patientenbefragung eine Bestandsaufnahme, inwieweit diese Qualitätsanforderungen in der Gesundheitsversorgung umgesetzt sind – wieder aus Sicht der Betroffenen. Entlang der Ergebnisse der Patientenbefragung können entsprechende Verbesserungsmaßnahmen in Krankenhäusern und Arztpraxen abgeleitet werden, die sich folglich an den Bedürfnissen und Bedarfen der Patientinnen und Patienten orientieren. Über das gezielte Erfragen von qualitätsrelevanten Ereignissen, Situationen oder Zuständen lassen sich konkrete Verbesserungsmaßnahmen für den Versorgungsalltag ableiten, die sich auf die allgemeine Ausgestaltung übergeordneter Strukturen und Prozesse beziehen. Insofern erfordert ein handlungsleitender Umgang mit den Ergebnissen der Patientenbefragung ein ausgeprägtes Verständnis von Qualitätsmanagement. So können Abläufe beleuchtet und Verbesserungsmöglichkeiten erkannt und etabliert werden.

Insgesamt ist zu konstatieren, dass die Etablierung einer Patientenbefragung, die Einfluss auf eine patientenzentrierte Versorgungsqualität hat, auch Aufwände mit sich bringt. Diese lohnen sich, da es dadurch möglich ist, deutschlandweit Einblicke in die Versorgungsqualität aus Patientensicht zu erhalten und so flächendeckend die Versorgung im Sinne der Patientenzentrierung auszugestalten. Die externe Qualitätssicherung bietet die Möglichkeit, über den regelhaften Einsatz von Patientenbefragungen den Gedanken und die praktische Umsetzung einer patientenzentrierten Versorgung mehr und mehr zu etablieren. Die ersten Anfänge sind seit 2022 erfolgt. Nun gilt es, davon noch weiter Gebrauch zu machen.

## Footnotes

1 Der verpflichtende Einsatz basiert auf der Richtlinie zur datengestützten einrichtungsüberreifenden Qualitätssicherung (DeQS-RL) des G‑BA.

2 Die Auswertung bezog sich auf 136 Patientinnen und Patienten aus 19 Fokusgruppen zu 4 Entwicklungen zu Patientenbefragungen mit den Themen PCI/Koronarangiografie, Dialyse/Nierentransplantation, ambulante Psychotherapie und Schizophrenie.

3 Im Rahmen der gesetzlichen Qualitätssicherung wurden für das Jahr 2024 insgesamt 795.541 PCI und Koronarangiografien bei gesetzlich versicherten Patientinnen und Patienten registriert. Aus dieser Grundgesamtheit wurde für die Patientenbefragung je Krankenhaus bzw. Arztpraxis eine Zufallsstichprobe gezogen.

4 Siehe auch Angebot der Bundeszahnärztekammer zu Teach-Back-Techniken: https://www.bzaek-teach-back.de/, Zugriff am 11.11.2025.

5 Siehe Beauftragung des Gemeinsamen Bundesausschusses vom 07.05.2025: https://www.g-ba.de/downloads/39-261-7198/2025-05-07_DeQS-RL_IQTIG-Beauftragung_Umsetzung-Patientenbefragung-QS-PCI.pdf, Zugriff am 11.11.2025.

## References

[1] Institute of Medicine (2001) Crossing the Quality Chasm. In: A New Health System for the 21st Century. National Academies Press, Washington 25057539

[2] Scholl I, Zill JM, Härter M, Dirmaier J (2014) An integrative model of patient-centeredness—a systematic review and concept analysis. Plos One 9:e107828. 10.1371/journal.pone.0107828 25229640 10.1371/journal.pone.0107828 PMC4168256

[3] Elwyn G, Durand MA, Song J et al (2017) A three-talk model for shared decision making: multistage consultation process. BMJ 359:j4891. 10.1136/bmj.j4891 29109079 10.1136/bmj.j4891 PMC5683042

[4] Bomhof-Roordink H, Fischer MJ, van Duijn-Bakker N et al (2019) Shared decision making in oncology: A model based on patients’, health care professionals’, and researchers’ views. Psychooncology 28:139–146. 10.1002/pon.4923 30346076 10.1002/pon.4923

[5] Lloyd H, Jenkinson C, Hadi M, Gibbons E, Fitzpatrick R (2014) Patient reports of the outcomes of treatment: a structured review of approaches. Health Qual Life Outcomes 12:5. 10.1186/1477-7525-12-5 24422873 10.1186/1477-7525-12-5 PMC3899626

[6] Cleary PD (1999) The increasing importance of patient surveys. Now that sound methods exist, patient surveys can facilitate improvement. BMJ 319:720–721. 10.1136/bmj.319.7212.720 10487981 10.1136/bmj.319.7212.720 PMC1116581

[7] Gerteis M, Edgman-Levitan S, Daley J (1993) Through the patient’s eyes. In: Understanding and promoting patient-centered care. Jossey-Bass, San Francisco

[8] Friedel AL, Siegel S, Kirstein CF et al (2023) Measuring Patient Experience and Patient Satisfaction-How Are We Doing It and Why Does It Matter? A Comparison of European and U.S. American Approaches. Healthcare. 10.3390/healthcare11060797 36981454 10.3390/healthcare11060797 PMC10048416

[9] (2025) https://iqtig.org/downloads/berichte/2025/IQTIG_BQB-2025_2025-11-07.pdf. Zugegriffen: Institut für Qualitätssicherung und Transparenz im Gesundheitswesen

[10] Bull C, Teede H, Watson D, Callander EJ (2022) Selecting and Implementing Patient-Reported Outcome and Experience Measures to Assess Health System Performance. Jama Health Forum 3:e220326. 10.1001/jamahealthforum.2022.0326 36218960 10.1001/jamahealthforum.2022.0326

[11] Yen PH, Leasure AR (2019) Use and Effectiveness of the Teach-Back Method in Patient Education and Health Outcomes. Fed Pract 36:284–289 31258322 PMC6590951

[12] (2024) https://iqtig.org/downloads/berichte/2024/IQTIG_Methodische-Grundlagen_Version-2.1_2024-11-27.pdf. Zugegriffen: 2003

[13] Klein S, Porst R (2000) https://www.gesis.org/fileadmin/upload/forschung/publikationen/gesis_reihen/gesis_methodenberichte/2000/00_10.pdf. Zugegriffen: 2001

[14] Schnell R (2019) Survey-Interviews. In: Methoden standardisierter Befragungen. Springer, Wiesbaden

